# Lower limb biomechanics and knee tissue loading under fatigue during the badminton forward lunge in recreational female players

**DOI:** 10.3389/fpubh.2026.1936430

**Published:** 2026-09-04

**Authors:** Jiankai Chen, Yan Zhang, Maosheng Ye, Gusztáv Fekete, Yaodong Gu

**Affiliations:** 1 Faculty of Sport Science, Ningbo University, Ningbo, China; 2 Audi Hungária Faculty of Engineering, Széchenyi István University, Győr, Hungary

**Keywords:** fatigue, forehand forward lunge, knee joint loading, lower limb biomechanics, recreational female players

## Abstract

**Background:**

Badminton is widely promoted as a lifelong physical activity, yet repetitive forward lunges performed under fatigue may increase the risk of knee injury, particularly among recreational female players. Although fatigue-related changes in lower-limb biomechanics have been reported, their influence on tissue-level knee loading remains unclear. This study investigated the effects of fatigue on lower-limb biomechanics and internal knee loading during the badminton forehand forward lunge.

**Methods:**

A within-subject repeated-measures experimental design was used. Sixteen recreational female badminton players performed standardized forehand forward lunges before and after a fatigue protocol. Three-dimensional lower limb kinematics and kinetics were collected using a motion capture system and force platform. Time-varying biomechanical differences across the stance phase were analyzed using one-dimensional Statistical Parametric Mapping. A subject-specific finite element model was developed to evaluate stress and strain in the patellofemoral cartilage, menisci, and anterior cruciate ligament under the peak vertical ground reaction force (vGRF) loading condition.

**Results:**

Fatigue induced alterations in lower limb biomechanics throughout the stance phase (%), including reduced knee flexion (2.6–16.4%, *p* = 0.02, 54.8–65.7%, *p* = 0.03) and ankle dorsiflexion (0.0–36.2%, *p* < 0.01) with increased ankle eversion (69.0–93.3%, *p* < 0.01). At the peak vGRF instant, hip abduction decreased (*p* < 0.01), whereas knee varus angle (*p* = 0.02) and internal valgus moment increased (*p* = 0.04). Finite element analysis demonstrated greater loading of passive knee tissues after fatigue, with patellar cartilage contact pressure increasing by 16.4%, patellar cartilage von Mises stress by 19.3%, medial meniscal stress by 5.5%, and anterior cruciate ligament (ACL) stress and maximum principal strain by 7.4 and 18.4%, respectively. In addition, the regions of peak ACL stress and strain shifted toward the femoral attachment after fatigue.

**Conclusion:**

Fatigue altered lower limb biomechanics and increased passive knee tissues during the badminton forehand forward lunge in recreational female players, indicating a less favorable mechanical environment for the knee under fatigued condition. Recognition of these biomechanical changes may help reduce knee injury risk and support long-term badminton participation among recreational female players.

## 1 Introduction

Badminton is a widely played racket sport that promotes physical activity and health among recreational participants. The forehand forward lunge is one of the most frequently performed and mechanically demanding techniques in badminton, requiring rapid forward acceleration followed by abrupt single leg deceleration. The short stance phase exposes the lower limb to substantial impact loading, which is considered an important contributor to musculoskeletal injury (1). Epidemiological studies have identified lunge as a major mechanism of lower limb injuries in badminton, with the knee being one of the most affected joints (2–4). Repeated execution of forward lunges can induce acute muscular fatigue, potentially reducing the ability to attenuate impact loads and altering lower limb movement strategies. In addition, female players exhibit different joint mechanics from males and may be more vulnerable to injury (5). Therefore, understanding fatigue-related biomechanical adaptations in recreational female players may provide valuable information for injury prevention.

Previous studies have examined fatigue-induced changes in lower limb biomechanics during badminton lunges. Fatigue has been associated with reduced knee flexion, increased leg stiffness, and altered joint moments during forehand and backhand forward lunges (6–8). Research involving amateur players further demonstrated that fatigue, lunge direction, and gender affect lower limb kinematics and kinetics, with females showing greater loading in selected variables under fatigued conditions (9). Functional data analysis also suggested that fatigue may produce a “top-down” compensatory response, characterized by reduced hip and knee motion and increased ankle motion during lunging tasks among elite male badminton players (10). These findings indicate that fatigue-related biomechanical responses are influenced by participant characteristics and task demands. However, changes in time-varying lower limb biomechanics throughout the badminton forward lunge remain insufficiently understood in recreational female players.

The knee plays an essential role in impact attenuation and lower limb stability during lunging. Previous investigations have provided detailed information regarding knee biomechanics in badminton through approaches such as dual fluoroscopic imaging, wearable-based prediction models, and finite element (FE) analysis (11–13). These studies have demonstrated that lunge conditions affect knee motion, joint loading, and internal contact mechanics. However, existing research has primarily focused on external joint measures or non-fatigue conditions. The effects of fatigue on internal knee tissue loading, including cartilage, meniscal, and ligament responses, remain unclear.

Therefore, this study combined one-dimensional Statistical Parametric Mapping (SPM1d) with a representative subject-specific FE model to investigate the biomechanical response to fatigue during the forehand forward lunge in recreational female badminton players. Continuous lower limb kinematics and kinetics were evaluated throughout the stance phase, and corresponding knee loading conditions were incorporated into the FE model to estimate stresses and strains. We hypothesized that fatigue would alter lower limb kinematics and kinetics throughout the stance phase and increase knee tissue loading. The findings may provide biomechanical evidence for understanding fatigue-related tissue loading during badminton lunges and inform injury prevention strategies.

## 2 Materials and methods

### 2.1 Participants

An *a priori* power analysis was performed using G*Power software (version 3.1.9.7; Heinrich Heine University of Düsseldorf, Düsseldorf, Germany). For a paired-samples t-test, an effect size of dz = 0.8, an alpha level of 0.05, and a statistical power of 0.80 indicated that a minimum sample size of 15 participants was required (14, 15). Accordingly, 16 recreational female badminton players from Ningbo University were recruited for this study. The participants were 20.83 ± 1.47 years old, with a height of 165.00 ± 3.81 cm and a body mass of 56.47 ± 6.49 kg. Participants were eligible for inclusion if they were female non-professional badminton players aged 18–25 years, were right-leg dominant, had at least 2 years of badminton playing experience, trained for a minimum of 6 h per week, had no formal competition experience, and had no history of lower-limb injury within the previous year (5, 9, 16). Before data collection, all participants received written information regarding the study objectives, experimental procedures, and testing requirements. Written informed consent was obtained from all participants. The study protocol was approved by the Ningbo University Human Ethics Committee (TY2025066).

### 2.2 Data collection

This study used a within-subject repeated-measures experimental design. Before testing, participants’ height and body mass were measured. Leg length was defined as the vertical distance from the right anterior superior iliac spine to the ground (17). The target lunge distance was set at 1.5 times the individual leg length. A shuttlecock was suspended at a horizontal distance of 2.0 times the leg length from the starting position and at a height corresponding to 70% of the participant’s standing height. Participants completed a 10-min jogging warm-up at 7 km/h before data collection. All participants wore tight-fitting athletic clothing and unified badminton shoes during testing (18).

**Figure 1:**
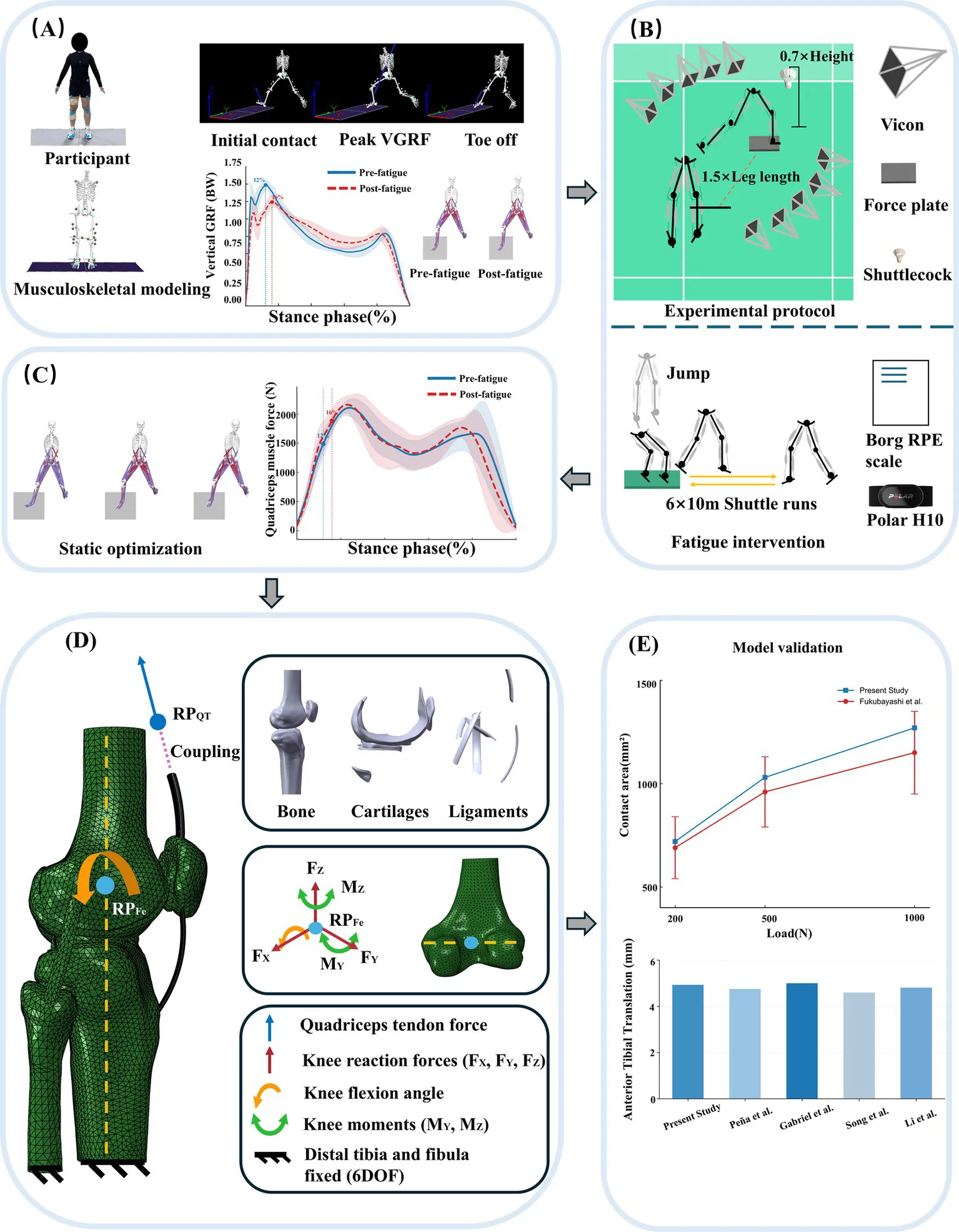
Experimental workflow and finite element modeling. **(A)** Data collection. **(B)** Experimental setup and fatigue protocol. **(C)** Muscle force calculation. **(D)** Boundary and loading conditions. **(E)** Model validation.

A total of 38 retroreflective markers were attached to anatomical landmarks according to the OpenSim Gait2392 model (Figure 1A). Three-dimensional (3D) motion data were collected using a 10-camera Vicon motion capture system at 200 Hz (MX-T20, Oxford Metrics Ltd., Oxford, UK). Ground reaction force data were simultaneously collected at 1000 Hz using a floor-embedded force plate (Kistler Instruments AG, Winterthur, Switzerland). Initial contact was defined as the instant when the vertical ground reaction force (vGRF) exceeded 20 N. The forehand forward lunge was defined as a forward movement toward the racket side, with the trunk facing the net, followed by rapid recovery to the starting position (19). A certified badminton coach supervised the testing procedure and confirmed the validity of each trial. Three valid pre-fatigue trials were collected, with a 30-s rest interval between trials.

### 2.3 Fatigue protocol

After the pre-fatigue trials, participants performed a fatigue protocol based on the procedure described by Fang et al. (10). Each cycle consisted of 6 × 10 m shuttle runs followed immediately by five consecutive maximal vertical jumps (Figure 1B). The cycles were repeated until fatigue was reached. Fatigue was identified when any one of the following criteria was met: the average height of the five consecutive vertical jumps decreased to below 70% of the individual pre-test maximal jump height, or the Borg Rating of Perceived Exertion score reached 17 or higher on the 6–20 scale. Heart rate was continuously monitored using a chest strap heart rate monitor (Polar H10 C77D1F25, Polar Electro, Finland). For participant safety, the protocol was stopped immediately if heart rate reached the age-predicted maximal value (220 – age). Immediately after the fatigue protocol, three valid post-fatigue forehand forward lunge trials were collected.

### 2.4 Muscle force calculation

The standard OpenSim musculoskeletal model was scaled to each participant’s anthropometric characteristics. Inverse kinematics was performed by minimizing the position errors between experimental and virtual markers. As shown in Figure 1C, static optimization was subsequently performed to minimize the sum of squared muscle activations and to estimate quadriceps forces throughout the stance phase (20). The tendon force at the instant of peak vGRF was extracted and used as an input for the FE simulations.

### 2.5 Finite element analysis

One participant whose anthropometric characteristics closely matched the cohort average underwent magnetic resonance imaging (MRI) of the right knee joint. 3D geometries of the knee joint structures were reconstructed from the imaging data. The four primary knee ligaments, including anterior cruciate ligament (ACL), posterior cruciate ligament (PCL), medial collateral ligament (MCL), and lateral collateral ligament (LCL), with the patellar tendon (PT) and quadriceps tendon (QT), were constructed in SolidWorks (version 2020; Dassault Systèmes, USA) based on anatomical attachment footprints identified from the MRI scans (21, 22).

The reconstructed models were imported into HyperMesh (version 2020; Altair Engineering, USA) for mesh generation. Bone, meniscus, cartilage, MCL, LCL, PT, and QT were meshed using 4-node linear tetrahedral elements (C3D4), whereas the ACL and PCL were meshed using 10-node quadratic hybrid tetrahedral elements (C3D10H). A mesh size was accepted when the relative difference in peak von Mises stress between two consecutive mesh refinements was less than 4% (23). Table 1 summarizes the final mesh sizes and element numbers.

**Table 1:** Element and material properties of components in the FE model.

Components		References	Element number	Mesh size (mm)	Model assumption	Material assignment			
						Young’s Modulus (MPa)	Poisson’s ratio (ν)	C1	D1
Bone	Femur	Song et al. (26)	35,525	3	Isotropic linear elasticity	12,000	0.3	\	\
	Tibia		33,965						
	Fibula		5,128						
	Patella		4,315						
Meniscus	Medial	LeRoux et al. Peña et al. (25, 27)	19,804	1	Isotropic linear elasticity	59	0.49	\	\
	Lateral		13,794						
Cartilage	Femoral	Li et al. (24)	79,055	1	Isotropic linear elasticity	5	0.46	\	\
	Medial tibial		8,466						
	Lateral tibial		8,728						
	Patellar		16,540						
	Tibiofibular		4,286						
Ligament	ACL	Chen et al. (21)	1,465	1.5	Neo-Hookean	\	\	5.08	0.00683
	PCL		1,717					6.06	0.0041
	MCL		3,679		Isotropic linear elasticity	60	0.3	\	\
	LCL		2,555			60	0.3		
Tendon	PT	Shen et al. (22)	2,783			60	0.3	\	\
	QT		2,155			60	0.3		

ACL, anterior cruciate ligament; LCL, lateral collateral ligament; MCL, medial collateral ligament; PCL, posterior cruciate ligament; PT, patellar tendon; QT, quadriceps tendon.

Material properties assigned to each tissue were obtained from previous studies (21, 22, 24–27) and are listed in Table 1. Bones, menisci, articular cartilage, MCL, LCL, PT, and QT were assumed to be homogeneous, isotropic, and linearly elastic materials. ACL and PCL were modeled as nearly incompressible hyperelastic materials using a Neo-Hookean constitutive model.

FE simulations were performed using Abaqus (version 2020; Dassault Systèmes, France). Surface-to-surface contact pairs were defined at the tibiofemoral, meniscofemoral, meniscotibial, and patellofemoral interfaces (13). A penalty-based hard contact formulation was used for normal contact behavior, and frictionless contact was assigned for tangential behavior (13, 28). The anterior and posterior horn attachments of the menisci were tied to the tibial plateau to maintain physiological meniscal fixation. Tie constraints were also applied at all cartilage-to-bone, ligament-to-bone, and tendon-to-bone attachment regions (29).

Simulations were conducted at the instant of vGRF. The distal ends of the tibia and fibula were fully constrained in all six degrees of freedom. A reference point was created at the midpoint of the femoral trans-epicondylar line coupling to the femur (RP_Fe_). The knee flexion angle was prescribed to the RP_Fe_, while the varus-valgus and internal-external rotation were unconstrained. The 3D joint reaction force and the joint moments in the varus-valgus and internal-external rotation directions were applied to the RP_Fe._ Another reference point was created at the proximal cross-section of the quadriceps tendon (RP_QT_) coupling to the proximal quadriceps tendon surface. The resultant quadriceps force was applied to the RP_QT_ along the anatomical line of action of the quadriceps tendon. The pre-fatigue and post-fatigue models differed only in the experimentally derived boundary and loading conditions, including the knee flexion angle, joint reaction force, joint moments, and quadriceps force. All boundary and loading conditions (Figure 1D) were applied within a single quasi-static analysis step using a ramped amplitude function.

### 2.6 Data processing and statistical analysis

Raw marker trajectories and ground reaction force data were imported into Visual3D (C-Motion Inc., Germantown, MD, USA) for processing. Kinematic and kinetic data were filtered using a fourth-order zero-lag low-pass Butterworth filter with cut-off frequencies of 12 Hz and 50 Hz, respectively (8). Joint kinematics and inverse dynamics were subsequently calculated. Custom Python scripts were used to convert the processed data into the format required for OpenSim analysis. All kinematic and kinetic variables were time-normalized to 100% of the stance phase.

Data normality was assessed using the Shapiro–Wilk test. Paired comparisons between pre-fatigue and post-fatigue conditions were performed using paired samples t-tests for normally distributed variables and Wilcoxon signed-rank tests for non-normally distributed variables. Effect sizes appropriate to the corresponding statistical tests were calculated to quantify the magnitude of fatigue-related differences. The corresponding 95% confidence intervals were reported. Statistical analyses of discrete variables were performed using SPSS (Version 25.0; IBM Corp., Armonk, NY, USA). Continuous waveform differences across the stance phase were assessed using SPM1d paired-samples t-tests (30) implemented in MATLAB R2018a (The MathWorks, Natick, MA, USA). Statistical significance was set at α = 0.05.

### 2.7 Model validation

The model was validated in terms of tibiofemoral contact mechanics and anterior knee laxity. First, the knee joint was positioned in full extension and subjected to axial compressive loads of 200, 500, and 1,000 N. The resulting medial and lateral tibiofemoral contact areas were calculated and compared with the *in vitro* experimental measurements reported by Fukubayashi and Kurosawa (31). Second, a 134 N anterior force was applied to the tibia with the knee maintained in full extension. The resulting anterior tibial translation was compared with previously published experimental and computational studies under similar loading conditions (26–28, 32).

## 3 Results

### 3.1 Model validation

As shown in Figure 1E, the predicted tibiofemoral contact areas under 200, 500, and 1,000 N compressive loads were 730, 1,030, and 1,260 mm^2^, respectively. Under a 134 N anterior tibial load, the model predicted an anterior tibial translation of 4.9 mm. These results support the mechanical validity of the present FE model.

### 3.2 Joint kinematics

At initial contact, fatigue was associated with reduced hip external rotation (*p* = 0.01, *d* = 0.79) and ankle dorsiflexion (*p* < 0.01, *d* = 0.90), accompanied by greater ankle external rotation (*p* = 0.02, *d* = 0.69). At peak vGRF, fatigue resulted in smaller hip abduction (*p* < 0.01, *d* = 1.23), greater knee varus (*p* = 0.02, *d* = 0.71), reduced hip external rotation (*p* = 0.03, *d* = 0.63), and reduced ankle dorsiflexion (*p* = 0.01, *d* = 0.76) (Tables 2, 3).

**Table 2:** Comparison of lower limb joint angles at initial contact before and after fatigue.

Joint angles (°)		Pre-fatigue	Post-fatigue	*p*-value	95% CI	Effect size
Hip	Flexion	58.59 ± 4.73	56.93 ± 5.15	0.31	[−1.75,5.08]	0.27
	Abduction	−22.79 ± 2.74	−20.96 ± 3.00	0.08	[−3.90,0.24]	0.49
	External rotation	−5.32 ± 7.33	−1.05 ± 5.22	0.01*	[−7.25,-1.29]	0.79
Knee	Flexion	−24.66 ± 5.35	−21.04 ± 7.93	0.06	[−7.49,0.25]	0.52
	Varus	1.43 ± 5.31	2.06 ± 4.41	0.51	[−2.62,1.36]	0.18
	External rotation	−3.06 ± 4.63	−6.63 ± 6.93	0.07	[−0.38,7.52]	0.50
Ankle	Dorsiflexion	10.64 ± 2.55	6.40 ± 3.36	<0.01*	[1.62,6.85]	0.90
	Eversion	−4.05 ± 1.21	−5.21 ± 2.12	0.11	[−0.28,2.62]	0.45
	External rotation	−4.65 ± 2.28	−6.85 ± 1.72	0.02*	[0.44,3.96]	0.69

Values are presented as mean ± standard deviation; *p < 0.05; 95% CI, 95% confidence intervals.

**Table 3:** Comparison of lower limb joint angles at peak vGRF instant.

Joint angles (°)		Pre-fatigue	Post-fatigue	*p*-value	95% CI	Effect size
Hip	Flexion	79.25 ± 7.99	79.98 ± 7.54	0.15	[−1.76,0.30]	0.38
	Abduction	−18.00 ± 2.67	−11.31 ± 5.00	<0.01*	[−10.51,-2.87]	0.93
	External rotation	−6.04 ± 8.03	−2.06 ± 6.60	0.03*	[−7.45,-0.50]	0.63
Knee	Flexion	−62.09 ± 7.82	−64.31 ± 8.26	0.22	[−1.53,5.97]	0.33
	Varus	4.08 ± 7.53	7.72 ± 4.58	0.02*	[−6.48,-0.80]	0.71
	Internal rotation	4.49 ± 2.52	3.45 ± 4.23	0.34	[−1.20,3.28]	0.26
Ankle	Dorsiflexion	7.57 ± 2.86	5.65 ± 3.06	0.01*	[0.52,3.32]	0.76
	Eversion	−3.46 ± 1.13	−4.47 ± 1.19	0.07	[−0.08,2.10]	0.51
	External rotation	−0.74 ± 1.84	−0.91 ± 1.81	0.81	[−1.36,1.70]	0.06

Values are presented as mean ± standard deviation; *p < 0.05; 95% CI, 95% confidence intervals.

**Figure 2:**
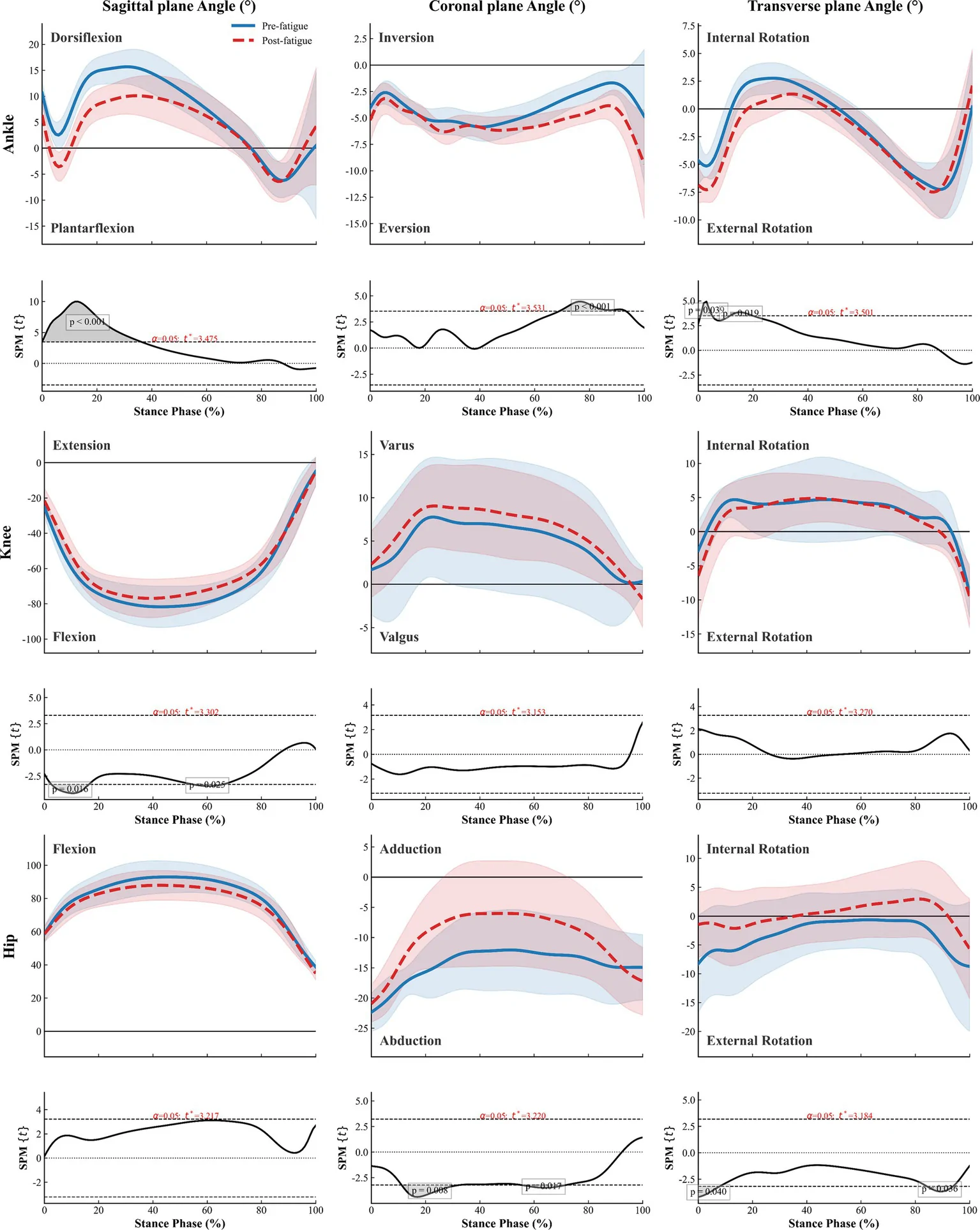
Lower limb joint angle waveforms and *SPM{t}* results during the stance phase.

SPM1d analysis revealed phase-specific differences throughout the stance phase (Figure 2). After fatigue, ankle dorsiflexion decreased during 0.0–36.2% of stance, whereas ankle eversion increased during 69.0–93.3%. Greater ankle external rotation was observed during 0.9–5.1% and 11.6–19.9% of stance. Knee flexion was reduced during 2.6–16.4% and 54.8–65.7% of stance. Hip abduction decreased during 11.2–32.4% and 55.3–71.7% of stance, while hip external rotation decreased during 0.0–7.9% and shifted toward greater internal rotation during 84.3–93.9% of stance.

### 3.3 Joint moments

At peak vGRF, fatigue increased the knee valgus moment (*p* = 0.04, *d* = 0.56) and reduced the ankle external rotation moment (*p* = 0.04, *d* = 0.56), whereas no significant changes were observed at the hip joint (Table 4).

**Table 4:** Comparison of lower limb joint moments at peak vGRF instant.

Joint moments (Nm/kg)		Pre-fatigue	Post-fatigue	*p*-value	95% CI	Effect size
Hip	Extension	−1.78 ± 0.49	−1.88 ± 0.59	0.46	[−0.19,0.40]	0.20
	Abduction	−0.00 ± 0.33	−0.25 ± 0.46	0.07	[−0.03,0.52]	0.50
	External rotation	−0.73 ± 0.28	−0.77 ± 0.30	0.65	[−0.14,0.21]	0.12
Knee	Extension	2.23 ± 0.55	2.09 ± 0.65	0.45	[−0.24,0.52]	0.19
	Valgus	−0.35 ± 0.20	−0.47 ± 0.18	0.04*	[0.00,0.25]	0.56
	Internal rotation	0.55 ± 0.20	0.57 ± 0.21	0.81	[−0.19,0.15]	0.06
Ankle	Plantarflexion	−0.66 ± 0.26	−0.71 ± 0.29	0.64	[−0.15,0.24]	0.12
	Eversion	−0.40 ± 0.12	−0.45 ± 0.10	0.12	[−0.01,0.11]	0.43
	External rotation	−0.34 ± 0.09	−0.29 ± 0.05	0.04*	[−0.09,-0.00]	0.56

Values are presented as mean ± standard deviation; *p < 0.05; 95% CI, 95% confidence intervals.

**Figure 3:**
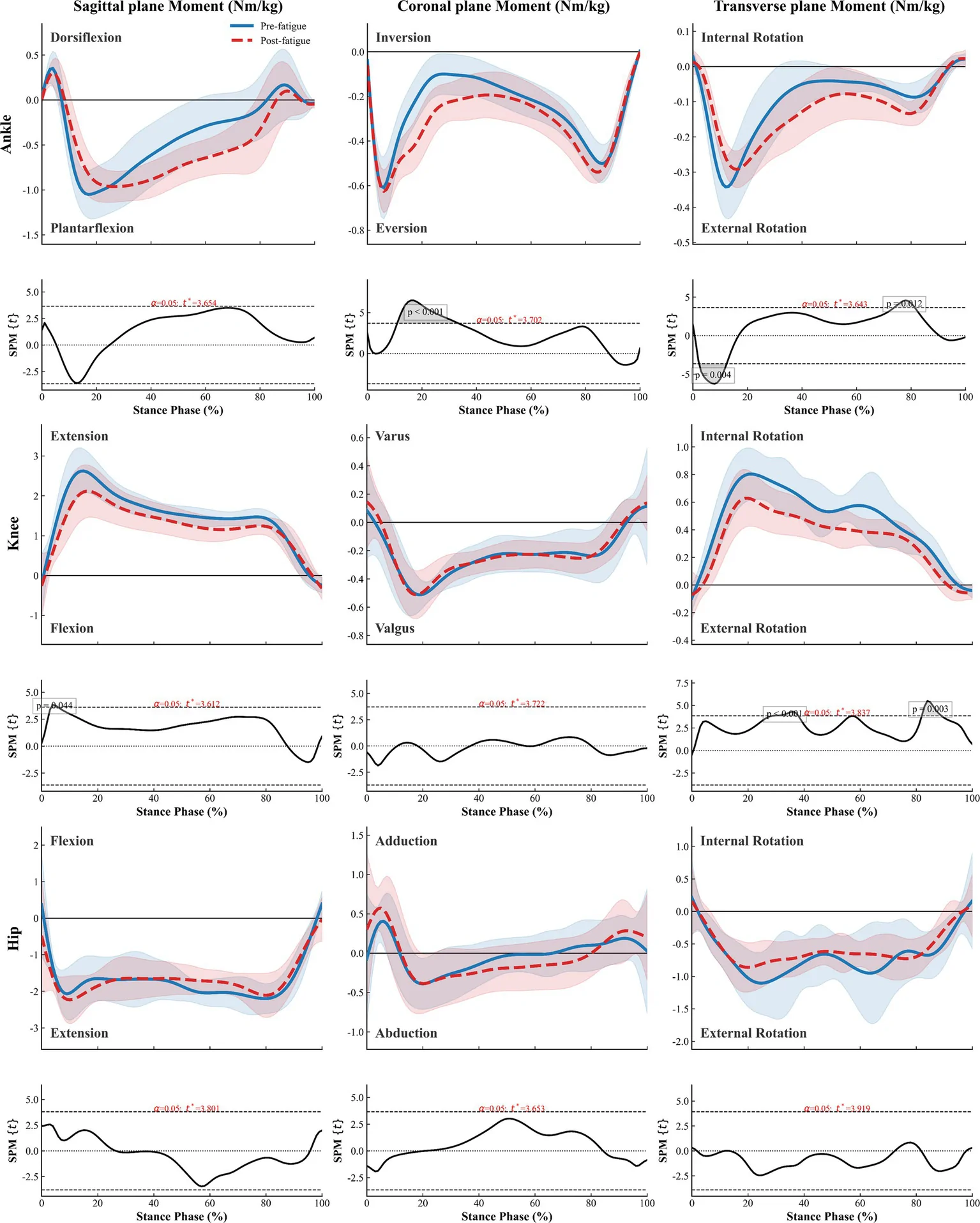
Lower limb joint moment waveforms and *SPM{t}* results during the stance phase.

SPM1d analysis revealed phase-specific alterations in joint moments during stance (Figure 3). Compared with the pre-fatigue condition, the post-fatigue condition exhibited reduced knee extension moment during 3.5–5.9% of stance and reduced knee internal rotation moment during 28.6–38.0% and 82.0–89.0% of stance. Ankle eversion moment increased during 10.9–33.3% of stance. The ankle external rotation moment showed a phase-dependent pattern, being lower during 2.3–12.4% but greater during 73.9–81.4% of stance after fatigue.

### 3.4 Meniscus von Mises stress

**Figure 4:**
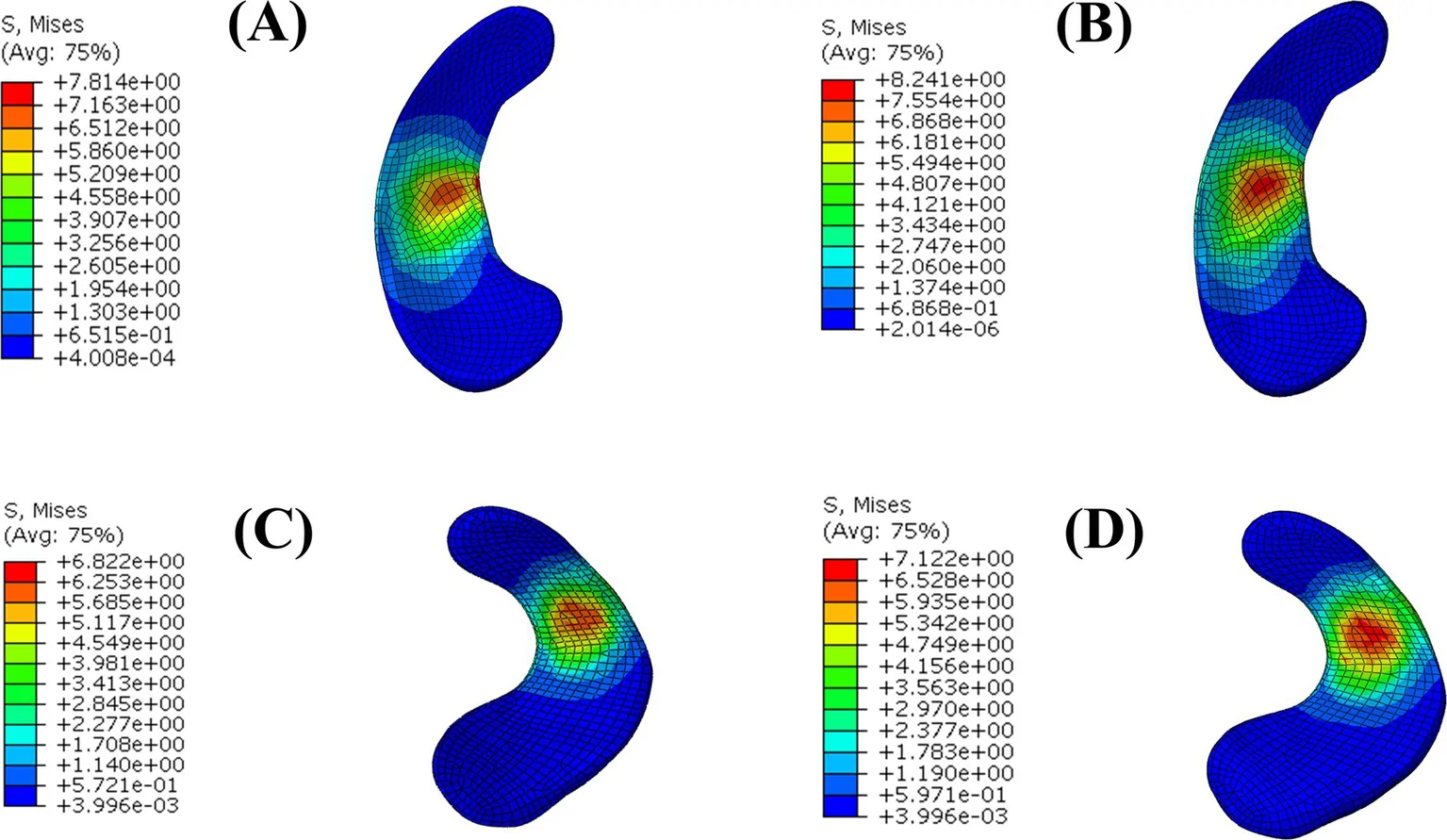
Von Mises stress distribution in the menisci at peak vGRF. **(A,B)** Medial meniscus under pre-fatigue and post-fatigue conditions, respectively; **(C,D)** lateral meniscus under pre-fatigue and post-fatigue conditions, respectively.

The spatial distribution of stress remained largely unchanged, although higher stress magnitudes were observed after fatigue. The highest von Mises stress was located in the medial meniscus under pre-fatigue and post-fatigue conditions (Figure 4). Fatigue increased the maximum von Mises stress in the medial meniscus from 7.81 to 8.24 MPa (+5.51%) and in the lateral meniscus from 6.82 to 7.12 MPa (+4.40%).

### 3.5 Patellofemoral joint loading

**Figure 5:**
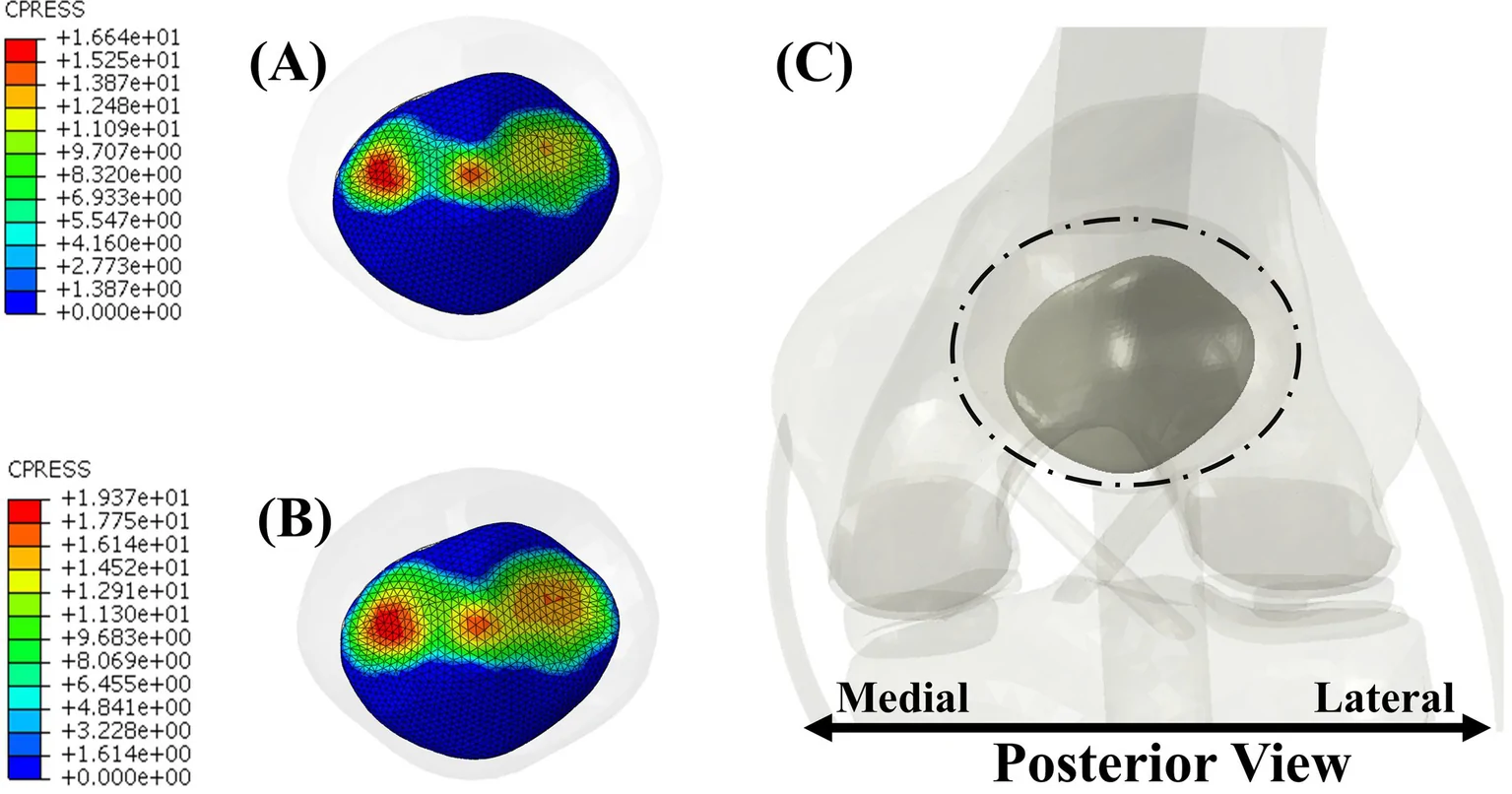
Patellar cartilage contact pressure at peak vGRF. **(A)** Pre-fatigue; **(B)** Post-fatigue; **(C)** Posterior view of the knee showing the medial-lateral orientation.

**Figure 6:**
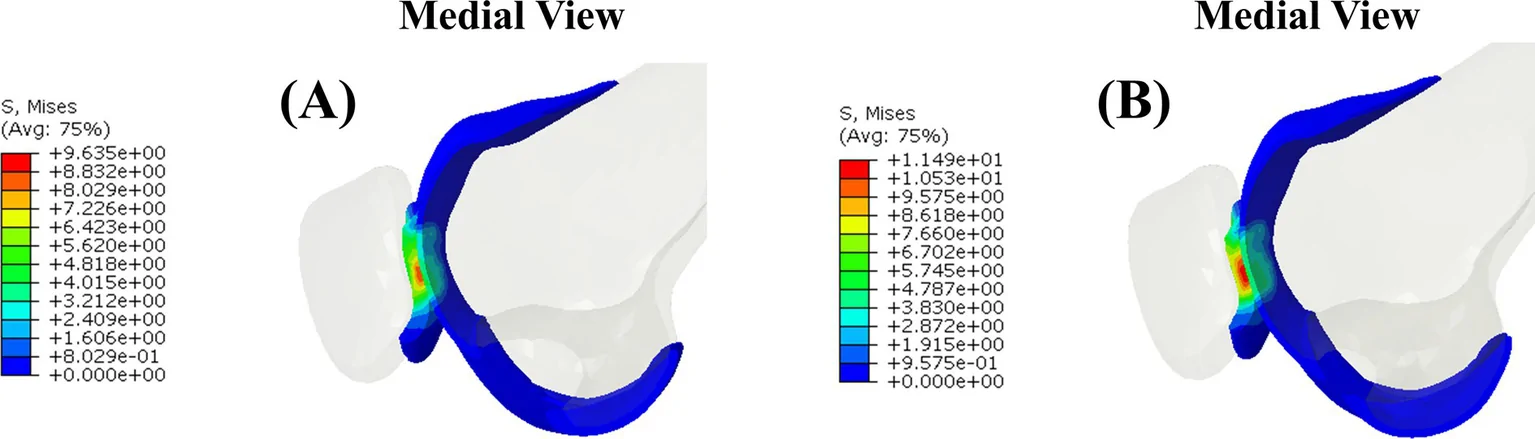
Von Mises stress distribution in the patellar cartilage at peak vGRF. **(A)** Pre-fatigue; **(B)** Post-fatigue.

Peak patellar cartilage contact pressure remained concentrated on the medial facet of the patella under both conditions (Figure 5). After fatigue, peak contact pressure increased from 16.64 to 19.37 MPa (+16.41%). Similarly, the maximum von Mises stress of the patellar cartilage increased from 9.64 to 11.49 MPa (+19.19%) (Figure 6). The high-pressure region was largely preserved despite the increase in loading magnitude.

### 3.6 ACL von Mises stress and principal strain

**Figure 7:**
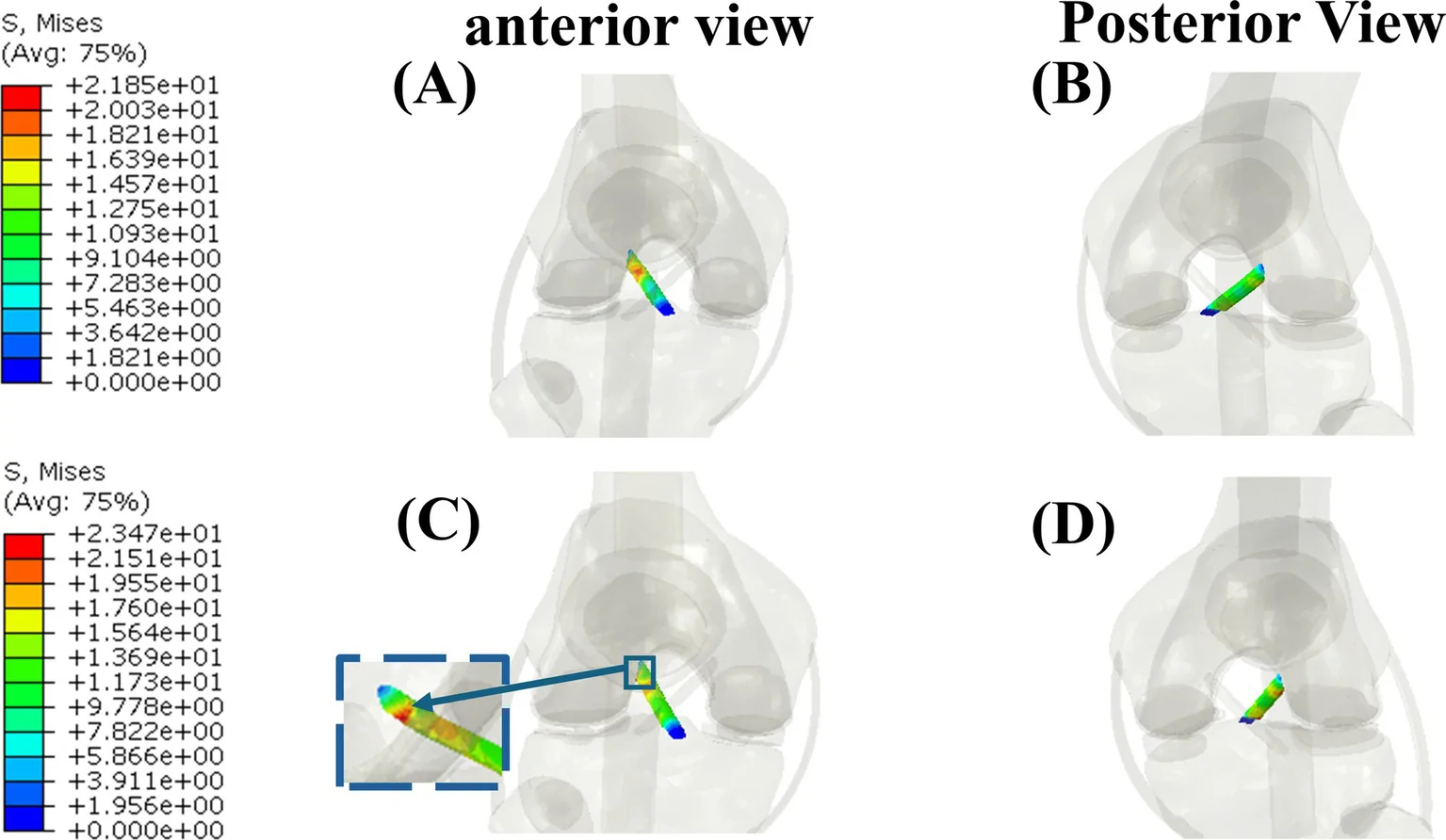
Von Mises stress distribution in the ACL at peak vGRF. **(A,B)** Pre-fatigue; **(C,D)** Post-fatigue. The inset in panel C highlights the proximal migration of the peak stress region after fatigue.

**Figure 8:**
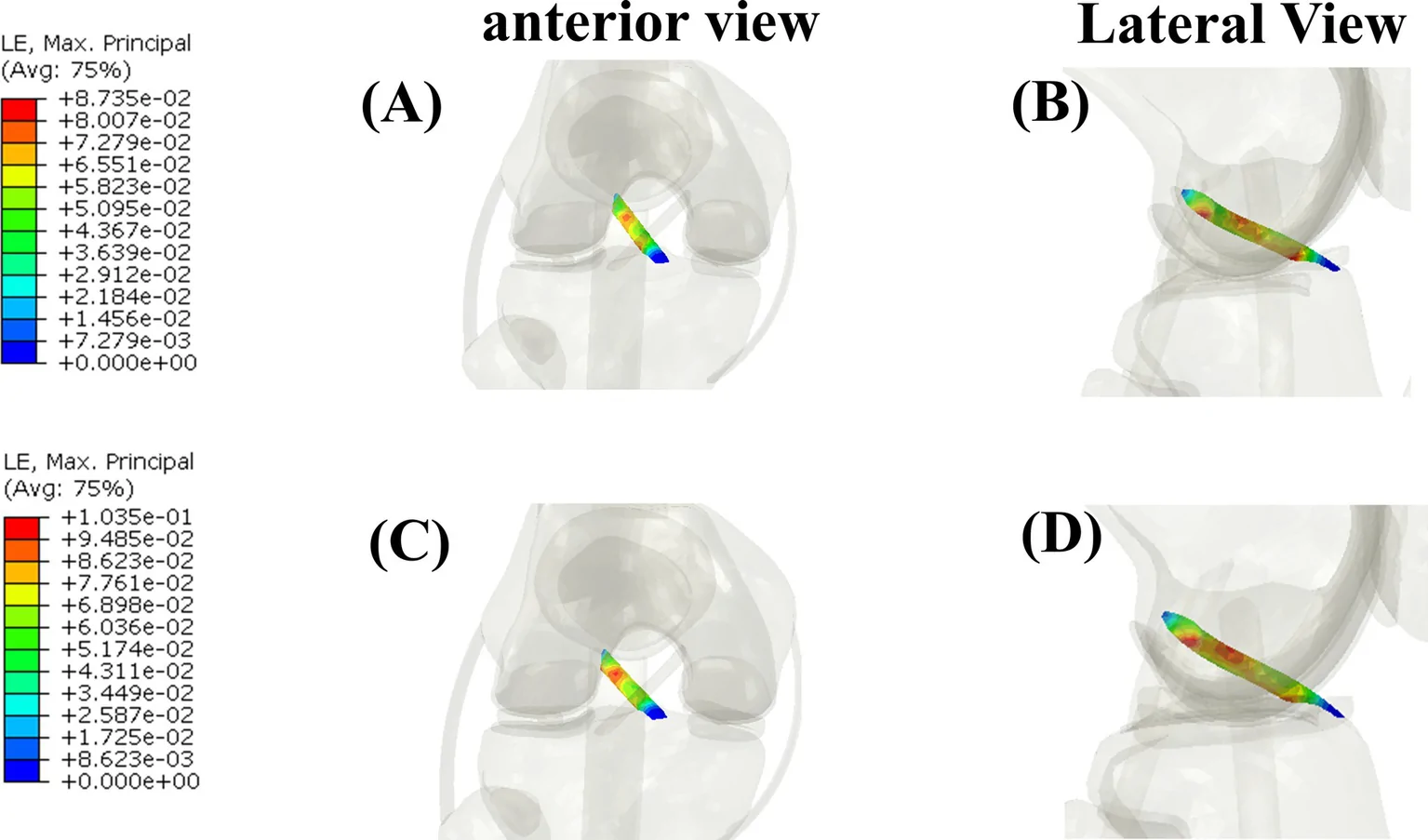
Maximum principal strain distribution in the ACL at peak vGRF. **(A,B)** Pre-fatigue; **(C,D)** post-fatigue.

The maximum von Mises stress of the ACL increased from 21.85 to 23.47 MPa (+7.41%) after fatigue (Figure 7). The region of peak stress shifted proximally and became concentrated near the femoral attachment. In addition, fatigue increased the maximum principal strain of ACL from 8.74 to 10.35% (Figure 8). The high strain region also shifted from the mid-proximal portion toward the proximal femoral attachment.

## 4 Discussion

This study combined motion analysis with subject-specific FE modeling to examine the biomechanical response to fatigue during the badminton forward lunge. The FE model was validated using previously reported experimental and computational data (26–28, 31, 32), providing a basis for interpreting the predicted tissue responses. Following fatigue, participants exhibited reduced knee flexion, greater knee varus, and altered ankle mechanics during the stance phase, which were accompanied by increased stress and contact pressure in the patellofemoral cartilage, menisci, and ACL, together with a proximal shift in ACL stress and strain concentration. These findings suggest that fatigue may limit the capacity to attenuate impact loading, resulting in greater reliance on passive joint tissues. Repeated exposure to this loading profile may contribute to cumulative tissue loading and could have implications for long-term joint health in recreational badminton players (33, 34).

The ankle dorsiflexion decreased at initial contact and gradually shifted to slight plantarflexion after fatigue. Additionally, the shift toward increased ankle plantarflexion with reduced knee flexion and hip external rotation during braking phase (early stance phase) suggests an altered braking strategy, with less joint excursion available to accommodate forward progression under fatigued conditions. Tong et al. (14) also reported increased ankle plantarflexion after dorsiflexor fatigue during the forehand lunge. In contrast to our findings, they observed increased knee flexion. This discrepancy may be related to the use of isolated dorsiflexor fatigue in their study compared with the badminton-specific fatigue protocol used in the present study. The increased ankle external rotation may indicate a tendency toward a toe-out foot rotation which has been associated with reduced ankle loading during badminton lunge landings (35) and may reflect an adaptive adjustment after fatigue. The increased ankle eversion moment and reduced external rotation moment further suggest a redistribution of multiplanar ankle loading. The support phase (mid stance phase) is critical in the forward lunge, as the loaded limb prepares for push-off. During this phase, fatigue reduced hip abduction and knee flexion, which may compromise the subsequent recovery movement. Previous evidence of reduced hip and knee ranges of motion after fatigue (10) complements the present phase-specific restriction in proximal joint motion. At the peak vGRF instant, reduced ankle dorsiflexion, hip abduction and external rotation accompanied by increased knee varus placed the knee in a less favorable configuration after fatigue. This may increase the demand for frontal plane stability and potentially increase strain on the lateral collateral ligament, which is sensitive to varus angulation (36). During push-off phase (late stance phase), fatigue mainly affected the ankle. The increased external rotation moment may increase ankle loading when initiating recovery from the lunge. Repeated lunges under fatigue may therefore increase the risk of ankle sprain.

Alterations in movement strategy were accompanied by changes in internal knee loading. Fatigue increased patellar cartilage stress and contact pressure. The increased quadriceps force after fatigue may have contributed to this response by increasing patellofemoral compression at the peak vGRF instant. Previous FE work has demonstrated higher patellofemoral loading with increased quadriceps force during lunge tasks (13). The elevated patellofemoral loading may increase susceptibility to patellofemoral pain, a common knee complaint in physically active women (37, 38). Meniscal stress also increased after fatigue, with a larger increase medially. The increased knee varus angle and internal knee valgus moment at peak vGRF are consistent with greater loading of the medial compartment. The varus alignment has been shown to increase medial compartment contact pressure (39), while cumulative adduction moment is associated with medial meniscus extrusion (40). This may account for the larger increase in medial meniscal stress after fatigue and potentially elevate the risk of overuse knee symptoms.

Following fatigue, there was an increase in ACL von Mises stress and maximum principal strain, with peak values shifting toward the femoral attachment. ACL loading is sensitive to knee flexion, and lower flexion angles can increase ACL stress and strain (41). In the present study, knee flexion was slightly increased after fatigue, while ACL stress and strain remained elevated. The FE model incorporated knee flexion angle, quadriceps tendon force, knee reaction forces and frontal and transverse moments. Simulated landing experiments showed increased ACL strain in response to knee abduction, anterior shear and internal rotation (42). The combined effects of knee posture and applied loads may therefore explain the increased ACL stress and strain despite the slightly greater knee flexion. The proximal concentration of stress and strain is clinically relevant because the femoral attachment is a common site of ACL rupture (43). Similar increases in internal tissue loading after fatigue have been reported in female badminton players during smash landing (44).

Several limitations should be acknowledged. First, although the sample size met the requirement of the *a priori* power analysis for within subject comparisons, the relatively small cohort may not fully capture the variability in biomechanical responses among recreational female badminton players. Studies with larger samples are needed to confirm the consistency and generalizability of these findings within this population. Second, the FE simulations were based on a representative subject-specific model. Consequently, the predicted tissue-level responses should be interpreted as mechanistic estimates rather than population-level outcomes. Nevertheless, this approach is consistent with many previous FE investigations that aimed to explore internal joint loading mechanisms. Third, the FE analysis was limited to the peak vGRF instant, whereas SPM identified biomechanical changes throughout the stance phase. Future dynamic FE analysis of the entire stance phase may clarify the relationship between fatigue-related biomechanical changes and tissue loading during the forward lunge. Fourth, foot orientation was not strictly standardized during the lunge task to preserve the natural movement patterns of recreational players. Although variations in foot placement may have influenced lower-limb biomechanics, excessive experimental control could have reduced the ecological validity of the task (11, 35). Finally, the laboratory fatigue protocol cannot fully replicate the intermittent and multidirectional demands of competitive badminton. However, the protocol successfully induced measurable biomechanical alterations and provided a controlled framework for examining fatigue-related mechanisms (10).

## 5 Conclusion

Fatigue altered lower limb joint kinematics, kinetics and internal loading of passive knee tissues during the badminton forehand forward lunge in recreational female players. By linking external biomechanics with tissue-level mechanical responses, this study provides additional insight into knee loading under fatigued conditions. For recreational female players, injury prevention should emphasize fatigue management and movement control during repeated lunges. Training may focus on maintaining hip and knee control during support, controlling ankle motion during braking, and improving ankle stability during push-off. These findings provide a biomechanical basis for developing training strategies aimed at limiting unfavorable loading during repeated lunges under fatigue.

## Data Availability

The raw data supporting the conclusions of this article will be made available by the authors, without undue reservation.
